# Reduction in Post-Marathon Peak Oxygen Consumption: Sign of Cardiac Fatigue in Amateur Runners?

**DOI:** 10.5935/abc.20150148

**Published:** 2016-02

**Authors:** Ana Paula Rennó Sierra, Anderson Donelli da Silveira, Ricardo Contesini Francisco, Rodrigo Bellios de Mattos Barretto, Carlos Anibal Sierra, Romeu Sergio Meneghelo, Maria Augusta Peduti Dal Molin Kiss, Nabil Ghorayeb, Ricardo Stein

**Affiliations:** 1 Escola de Educação Física e Esporte – Universidade de São Paulo, São Paulo, SP – Brazil; 2 Seção de Cardioesporte – Instituto Dante Pazzanese de Cardiologia, São Paulo, SP – Brazil; 3 Departamento de Medicina – Universidade Nove de Julho, São Paulo, SP – Brazil; 4 Grupo de Pesquisa em Cardiologia do Exercício do Hospital de Clínicas de Porto Alegre, Porto Alegre, RS – Brazil; 5 Serviço de Cardiologia do Hospital de Clínicas de Porto Alegre – Universidade Federal do Rio Grande do Sul, Porto Alegre, RS – Brazil; 6 Vitta Centro de Bem Estar Físico, Porto Alegre, RS – Brazil; 7 Setor de Eletrofisiologia – Instituto Dante Pazzanese de Cardiologia, São Paulo, SP – Brazil; 8 Setor de Ergometria e Reabilitação – Instituto Dante Pazzanese de Cardiologia, São Paulo, SP – Brazil; 9 Professor Adjunto II – Serviço de Cardiologia – Universidade Federal do Rio Grande do Sul, Porto Alegre, RS - Brasil; 10 Setor de Ecocardiografia – Instituto Dante Pazzanese de Cardiologia, São Paulo, SP – Brazil

**Keywords:** Exercise, Homeostasis, Sports, Ventricular Dysfunction, Running, Oxygen Consumption

## Abstract

**Background:**

Prolonged aerobic exercise, such as running a marathon, produces supraphysiological stress that can affect the athlete's homeostasis. Some degree of transient myocardial dysfunction ("cardiac fatigue") can be observed for several days after the race.

**Objective:**

To verify if there are changes in the cardiopulmonary capacity, and cardiac inotropy and lusitropy in amateur marathoners after running a marathon.

**Methods:**

The sample comprised 6 male amateur runners. All of them underwent cardiopulmonary exercise testing (CPET) one week before the São Paulo Marathon, and 3 to 4 days after that race. They underwent echocardiography 24 hours prior to and immediately after the marathon. All subjects were instructed not to exercise, to maintain their regular diet, ingest the same usual amount of liquids, and rest at least 8 hours a day in the period preceding the CPET.

**Results:**

The athletes completed the marathon in 221.5 (207; 250) minutes. In the post-marathon CPET, there was a significant reduction in peak oxygen consumption and peak oxygen pulse compared to the results obtained before the race (50.75 and 46.35 mL.kg^-1^ .min^-1^; 19.4 and 18.1 mL.btm, respectively). The echocardiography showed a significant reduction in the s' wave (inotropic marker), but no significant change in the E/e' ratio (lusitropic marker).

**Conclusions:**

In amateur runners, the marathon seems to promote changes in the cardiopulmonary capacity identified within 4 days after the race, with a reduction in the cardiac contractility. Such changes suggest that some degree of "cardiac fatigue" can occur.

## Introduction

Prolonged aerobic exercise, such as long-distance running events (marathon), causes supraphysiological stress that can affect the athlete's homeostasis. Depletion of energetic substrates, progressive body temperature increase, electrolyte imbalance and extensive muscle injury are common consequences of that practice^1^.

Transient myocardial dysfunction ("cardiac fatigue") can be observed over several days following prolonged exercise. It can be evidenced in healthy individuals by use of specific imaging tests and analysis of cardiac biomarkers in peripheral blood^2,3^. Transthoracic Doppler echocardiography performed after prolonged endurance exercise has shown changes in contractility, relaxation, and systolic and diastolic left ventricular function, such as changes in left ventricular internal diameter, an increase in A wave and a reduction in the E/A ratio^4,5^.

Impairment in pulmonary gas exchange and a reduction in pulmonary diffusing capacity observed after vigorous and prolonged aerobic exercise suggest that it can induce persistent respiratory functional impairment, even in the post-exercise period^3^. Stickland et al.^6^ have suggested that the change in pulmonary gas exchange following vigorous and prolonged exercise is influenced by the cardiovascular system. However, that relationship is yet to be established^6^.

Despite the increasing interest in the effects of a marathon on the cardiovascular and pulmonary systems, few studies using the cardiopulmonary exercise test (CPET) as a tool for that assessment have been published. Thus, this study aimed at assessing whether changes in the cardiopulmonary capacity of amateur athletes (pilot study) occur after a marathon.

## Methods

This is a case series of six athletes participating in the same São Paulo International Marathon edition, who were assessed at the Sports Cardiology outpatient clinic at Instituto Dante Pazzanese de Cardiologia. They signed the written informed consent, which, along with the study protocol, had been previously approved by the Committee on Ethics and Research of the institution, abiding by the rules of the Declaration of Helsinki.

This study included only male Caucasian runners participating in at least one marathon in the last five years and in a half marathon in the last year. All were healthy and reported no cardiopulmonary disease.

The first CPET was performed in the week preceding the marathon (3 days), and the second, 3 to 4 days after the event. The individuals refrained from training from the marathon day to the day of the second CPET after the marathon, and received written instructions to maintain a routine regarding liquid and food ingestion and rest over the post-marathon period. All exercise tests were performed on a treadmill (TEB^®^, APEX 200 model, São Paulo, Brazil), and the heart rate was obtained by using a TEB^®^ equipment (APEX 1000 model) and following a step protocol, initiating with an 8 km/h velocity and an 1 km/h increment every 1 minute, aiming at leading the individual to exhaustion.

For the expired gas analysis, a CardiO2 System analyzer (Medical Graphics Corporation^®^, Minnesota, USA) with sensors, allowing a breath-by-breath analysis, was used. The room conditions were controlled and maintained as follows: temperature, between 21°C and 23°C; relative air humidity registered in a thermo-hygrometer, 61%; and pressure registered in a Torricelli barometer, 703 mm Hg.

Peak exercise oxygen consumption (peak VO_2_) was quantified in the 30 seconds preceding the end of exertion and the 30 seconds after exertion. After data collection breath by breath, means were determined every 15 seconds, the highest value in the period being considered. In the last CPET stage, the runners pointed in the Borg scale, the value corresponding to their perception of exertion.

The athletes underwent echocardiography 3 days before the marathon and immediately after the event. The exam was performed in a tent located 100 meters after the finish line. The image acquisition time was of up to 10 minutes. A Vivid 7 device (GE Healthcare^®^, Milwaukee, WI, USA) with digital image storage capacity was used, equipped with a M4S sectorial transducer.

Complete echocardiographic study with single- and two-dimensional, conventional Doppler and tissue Doppler was performed according to the American Society of Echocardiography recommendations^7^. Echocardiography clips of four consecutive beats were captured. The tests were digitally stored and analyzed in a dedicated work station (ECHOPAC^®^ 6.0, Milwaukee, WI, USA).

### Statistical analysis

The SPSS software, version 22.0 (IBM Inc., Chicago IL, 2013) was used for statistical analysis. The results were expressed as medians and interquartile ranges, because the data were not normally distributed according to the Kolmogorov-Smirnov test. To assess the impact of the marathon on the two predetermined occasions (pre- and post-marathon), the nonparametric Wilcoxon test was used, and Spearman test was used to correlate the delta of the variables. The significance level adopted was 5%.

## Results

The São Paulo International Marathon was held in springtime, in a sunny day. The environmental conditions at the beginning of the race were as follows: temperature, 17.8°C; air humidity, 55%; and wind velocity, 1 m/s. At the end, they were 22.8°C, 59% and 2 m/s, respectively.

This pilot-study included six athletes. Table 1 shows their general characteristics, such as anthropometric data, age, race time, percentage of the peak velocity at which the runner did the marathon (mean velocity during the marathon divided by the peak velocity on the CPET multiplied by 100), and training volume (hours/week).

**Table 1:** General characteristics of the marathoners (n = 6)

**Variables**	**Median (25^th^ Perc.; 75^th^ Perc.)**
Age (years)	43.0 (36; 47)
Weight (kg)	67.1 (61; 75)
Height (cm)	164.5 (163; 168)
Race time (min)	221.5 (207; 250)
Mean velocity (km/h)	11.9 (9; 12)
Peak vel. %	64.0 (54; 66)
Training (hours/week)	10.0 (8; 13)

Data expressed as median and interquartile range; peak vel. %: mean velocity during the marathon divided by the peak velocity on the CPET multiplied by 100.

### Cardiopulmonary exercise test

All athletes showed good cardiopulmonary capacity, in both the pre- and post-marathon CPET, achieving practically the same peak velocity in both tests (medians of 18 and 18.5, respectively). All tests were maximum, with a respiratory quotient (R) greater than 1.1, and associated with intense fatigue, characterized by a Borg rating of perceived exertion scale of 19 or 20.

A significant reduction in VO_2_ peak and in peak oxygen pulse (PO_2_) was observed in post-marathon CPET. In addition, a reduction was observed in the ventilation/carbon dioxide production (VE/VCO_2_) slope, but with no difference in the maximum ventilation values between the tests. Table 2 shows the values of the CPET variables.

**Table 2:** Results of pre- and post-marathon cardiopulmonary exercise testing (n = 6)

**Variable**	**Pre-marathon**	**Post-marathon**	**p value**
Rest HR (bpm)	69 (65; 76)	70.5 (61; 80)	NS
Peak HR (bpm)	174 (167; 181)	175 (169; 186)	NS
Peak vel.(km/h)	18 (16; 20)	18.5 (17; 19)	NS
Peak VO_2_ (mL.kg^-1^.min^-1^)	50.75 (46; 52)	46.35 (43; 49)	*< 0.05
Peak VE (l/min)	134.2 (99; 148)	119.9 (111; 147)	NS
R	1.15 (1.10 1.19)	1.14 (1.11; 1.17)	NS
O_2_ pulse (ml.btm)	19.4 (17; 21)	18.1 (15; 19)	*< 0.05
Peak PetO_2_	101.5 (94.5; 105.7)	101.5 (96.5; 104.2)	NS
VE/VCO_2_ slope	33.7 (30; 41)	31.1 (27; 39)	*< 0.05

Data expressed as median and interquartile range (25^th^ percentile; 75^th^ percentile);

* p < 0.05 = significant; HR: heart rate; vel.: velocity; VO_2_. oxygen consumption; VE: ventilation; R: respiratory quotient; O_2_ pulse: oxygen pulse; PetO_2_: end-tidal O_2_ tension; VE/VCO_2_ slope; ratio between the inclination of the ventilation curve and the carbon dioxide production curve.

### Echocardiography

On echocardiography, the athletes showed ejection fraction within the normal range, but they differed at rest after the marathon, mainly due to heart rate increase.

Regarding the echocardiographic variables, a significant reduction in the values of E wave, s' wave and E/A ratio, as well as a concomitant increase in heart rate were observed (Table 3).

Cardiac lusitropy was assessed by use of the E/e' ratio, which showed no significant change after the marathon. Cardiac inotropy was assessed by use of the s' wave, which represents myocardial contractility. The s' wave showed a significant reduction after the marathon, suggesting a reduction in contractility, and, thus, in cardiac inotropy.

**Table 3:** Echocardiographic results before and immediately after the marathon (n = 6)

**Variable**	**Pre-marathon**	**Post-marathon**	**p value**
HR	62 (60; 67)	104 (101; 111)	*< 0.05
Systolic volume	88.5 (78.5; 100.1)	61 (50.7; 67.7)	*< 0.05
Cardiac output	5354 (4747; 6458)	6234 (5238; 7433)	NS
LVEDD	50.5 (48.5; 52.25)	51 (44.5; 57.7)	NS
LVESD	31.5 (29.2; 32.2)	32 (27.5; 34)	NS
EF	67.1 (65.5; 69.7)	61.6 (61; 67)	NS
E wave	0.9 (0.67; 1.02)	0.6 (0.5; 0.72)	*< 0.05
A wave	0.65 (0.47; 0.9)	0.9 (0.8; 0.92)	NS
E/A ratio	1.33 (1.08; 1.54)	0.7 (0.6; 0.79)	*< 0.05
s' wave	8.8 (8.2; 9.7)	6.7 (5.9; 8)	*< 0.05
e' wave	9.2 (8.4; 10.6)	8.5 (6.4; 10.4)	NS
a' wave	8.1 (7.6; 9.1)	7.6 (6.6; 9.6)	NS
E/e' ratio	0.09 (0.08; 0.1)	0.08 (0.06; 0.09)	NS

Data expressed as median and interquartile range (25^th^ percentile; 75^th^ percentile);

* p < 0.05 = significant; HR: heart rate; LVEDD: left ventricular end-diastolic diameter; LVESD: left ventricular end-systolic diameter; EF: ejection fraction.

## Discussion

The major finding in this study was the significant reduction in post-marathon peak VO_2_ as compared to the pre-marathon period. This happened although the athletes maintained the same performance on CPET, meaning that they reached the same mean maximum velocity in both tests.

Some changes identified in our study are in accordance with the results of previous studies carried out in athletes completing endurance events. Kasikcioglu et al.,^2^ in a study published in 2006, reported a reduction in peak VO_2_ and suggested that it could have resulted from a deceleration in the muscle oxygen kinetics after prolonged exercise^2^. Similarly, Miles et al.^8^ have reported a reduction in pulmonary diffusing capacity after prolonged exercise, and that persisted for a a certain amount of time. However, the changes in the system of oxygen delivery to tissues occurring after exercise have not been clarified.

In that context, the post-marathon drop in peak VO_2_ and PO_2_ can suggest some degree of "cardiac fatigue". With the PO_2_ drop on the post-marathon test, the reduction in cardiac inotropism seems to play a significant role in the VO_2_ drop in that same period. This might have been the factor contributing most to VO_2_ drop, because the peak heart rate was similar in both tests. In addition, despite the partial benefit demonstrated by pulmonary compensation, with greater ventilatory efficiency and optimization in O_2_ use to produce the same exertion, a reduction in cardiac output is evidenced in the post-marathon period. Based on those findings, one can speculate a worsening of myocardial performance after the marathon stress.

Those hypotheses are supported by both echocardiographic and CPET post-marathon findings. The modifications in contractility on echocardiography, along with the drop in peak PO_2_ and peak VO_2_ on CPET, support the hypothesis that athletes can have some degree of "cardiac fatigue", mainly due to the changes in cardiac inotropism, with no change in lusitropy. However, the variables of diastolic function assessment had no change.

The VE/VCO_2_ slope showed a significant reduction from the pre-marathon to the post-marathon period. No plausible explanation for this finding was found, but the small absolute variation of that variable makes us believe that there is no physiological relevance for that difference.

This study's limitations include the small and selected sample (all Caucasian males of similar ages), which can have caused a bias in the results obtained, in addition to hindering any extrapolation regarding external validity. It is worth noting the lack of control regarding the previous training of the athletes. Similarly, there was no control about possible interventions performed in the recovery period (3-4 days), which can have interfered with the results observed (water ingestion, food and resting time). New studies are required to support our findings.

## Conclusions

In amateur athletes, marathon seems to cause changes in the cardiopulmonary capacity identified up to four days after the event, with a reduction in contractility, and, thus, in cardiac inotropy. Such modifications suggest that some degree of "cardiac fatigue" can occur.

## References

[1] Coyle EF (2007). Physiological regulation of marathon
performance. Sports Med.

[2] Kasikcioglu E, Arslan A, Topcu B, Sayli O, Akhan H, Oflaz H (2006). Cardiac fatigue and oxygen kinetics after prolonged
exercise. Int J Cardiol.

[3] Stickland MK, Anderson WD, Haykowsky MJ, Welsh RC, Petersen SR, Jones RL (2004). Effects of prolonged exercise to exhaustion on
left-ventricular function and pulmonary gas exchange. Respir Physiol Neurobiol.

[4] Whyte G, George K, Shave R, Dawson E, Stephenson C, Edwards B (2005). Impact of marathon running on cardiac structure and function
in recreational runners. Clin Sci (Lond).

[5] Wilson M, O'Hanlon R, Prasad S, Oxborough D, Godfrey R, Alpendurada F (2011). Biological markers of cardiac damage are not related to
measures of cardiac systolic and diastolic function using
cardiovascular magnetic resonance and echocardiography after an acute
bout of prolonged endurance exercise. Br J Sports Med.

[6] Stickland MK, Petersen SR, Haykowsky MJ, Taylor DA, Jones RL (2003). The effects of cycle racing on pulmonary diffusion capacity
and left ventricular systolic function. Respir Physiol Neurobiol.

[7] Lang RM, Badano LP, Mor-Avi V, Afilalo J, Armstrong A, Ernande L (2015). Recommendations for cardiac chamber quantification by
echocardiography in adults: an update from the American Society of
Echocardiography and the European Association of Cardiovascular
Imaging. J Am Soc Echocardiogr.

[8] Miles DS, Doerr CE, Schonfeld SA, Sinks DE, Gotshall RW (1983). Changes in pulmonary diffusing capacity and closing volume
after running a marathon. Respir Physiol.

